# Bis(μ-phenyl­methano­lato)bis­({4-[(*E*)-(4-*tert*-butyl­phen­yl)(2-pyrid­ylmethyl­imino)meth­yl]-3-methyl-1-phenyl-1*H*-pyrazol-5-olato}zinc(II))

**DOI:** 10.1107/S1600536809024544

**Published:** 2009-07-04

**Authors:** Mon-Wei Hsiao, Chu-Chieh Lin

**Affiliations:** a Department of Chemistry, National Chung Hsing University, Taichung 402, Taiwan, Republic of China

## Abstract

In the title centrosymmetric dimeric Zn^II^ complex, [Zn_2_(C_27_H_27_N_4_O)_2_(C_7_H_7_O)_2_], the Zn^II^ center is coordinated by two N atoms and one O atom of the ketiminate ligand and two bridging O atoms of the benzyl­alkoxy groups. The geometry around the Zn^II^ ions is distorted trigonal-bipyramidal.

## Related literature

For the potential applications of polyesters, see: Gref *et al.* (1994 ▶); Jeong *et al.* (1997 ▶). Many zinc complexes with various ligands are effective initiators/catalysts for the ring-opening polymerization (ROP) of cyclic esters, see: Chamberlain *et al.* (2001 ▶); Williams *et al.* (2003 ▶); Dechy-Cabaret *et al.* (2004 ▶); Chen *et al.* (2005 ▶); Wu *et al.* (2006 ▶); Huang *et al.* (2009 ▶); Hung *et al.* (2008 ▶). Tripodal tridentate ligand-supported zinc complexes have been used for the polymerization of lactides, see: Chisholm *et al.* (2000 ▶). Recently, a series of zinc alkoxides (Yu *et al.*, 2002 ▶; Lee *et al.*, 2007 ▶) coordinated with simple *N*,*N*,*O*-tridentate ketiminate ligands has been synthesized and these derivatives showed highly catalytic activity with regard to the ROP of lactides. For Zn—O and Zn—N distances in other zinc ketiminate complexes, see: Hung & Lin (2009 ▶).
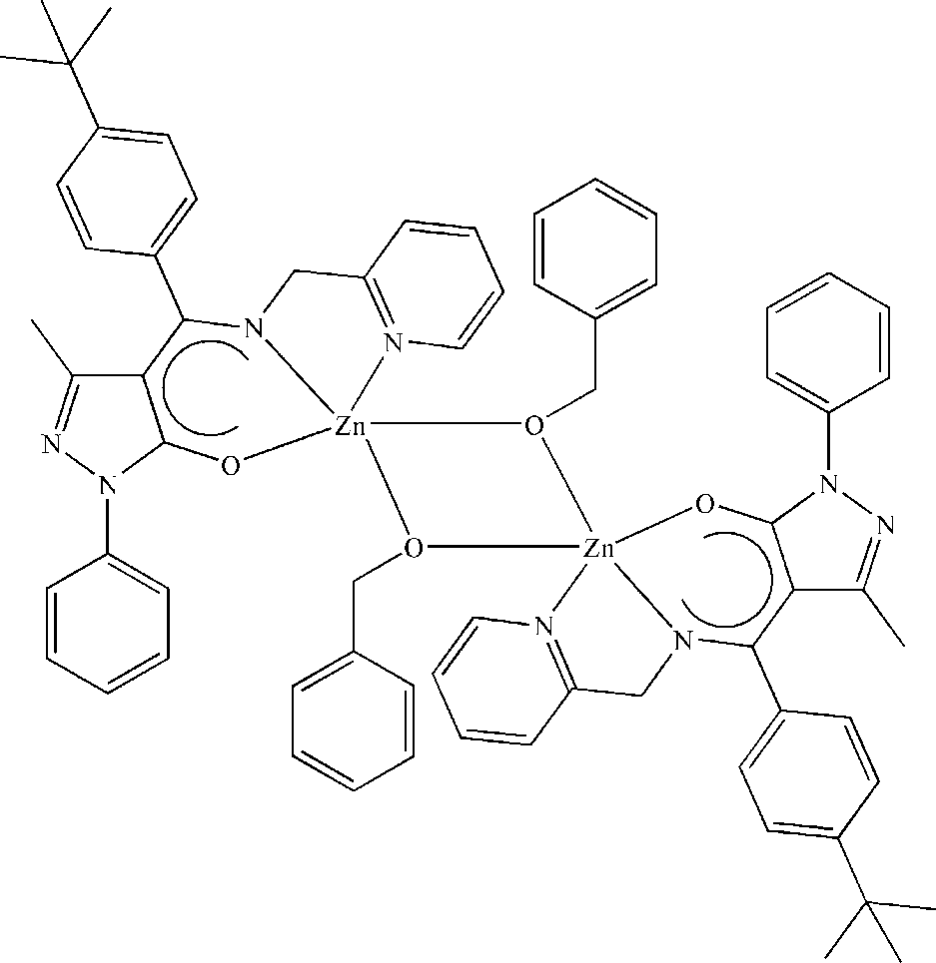


## Experimental

### 

#### Crystal data

[Zn_2_(C_27_H_27_N_4_O)_2_(C_7_H_7_O)_2_]
*M* *_r_* = 1192.04
Triclinic, 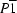

*a* = 9.0873 (15) Å
*b* = 13.363 (2) Å
*c* = 13.397 (2) Å
α = 72.206 (3)°
β = 74.018 (3)°
γ = 88.664 (3)°
*V* = 1485.9 (4) Å^3^
*Z* = 1
Mo *K*α radiation
μ = 0.86 mm^−1^
*T* = 293 K
0.41 × 0.32 × 0.25 mm

#### Data collection

Bruker SMART 1000 CCD diffractometer
Absorption correction: multi-scan (*SADABS*; Sheldrick, 1996 ▶) *T*_min_ = 0.719, *T*_max_ = 0.813
8258 measured reflections
5792 independent reflections
4450 reflections with *I* > 2σ(*I*)
*R*_int_ = 0.031

#### Refinement

*R*[*F* ^2^ > 2σ(*F* ^2^)] = 0.048
*wR*(*F* ^2^) = 0.137
*S* = 1.01
5792 reflections
370 parameters
H-atom parameters constrained
Δρ_max_ = 0.58 e Å^−3^
Δρ_min_ = −0.44 e Å^−3^

### 

Data collection: *SMART* (Bruker, 1999 ▶); cell refinement: *SAINT* (Bruker, 1999 ▶); data reduction: *SAINT*; program(s) used to solve structure: *SHELXS97* (Sheldrick, 2008 ▶); program(s) used to refine structure: *SHELXL97* (Sheldrick, 2008 ▶); molecular graphics: *SHELXTL* (Sheldrick, 2008 ▶); software used to prepare material for publication: *SHELXTL*.

## Supplementary Material

Crystal structure: contains datablocks global, I. DOI: 10.1107/S1600536809024544/bt2979sup1.cif

Structure factors: contains datablocks I. DOI: 10.1107/S1600536809024544/bt2979Isup2.hkl

Additional supplementary materials:  crystallographic information; 3D view; checkCIF report

## Figures and Tables

**Fig. 1.**
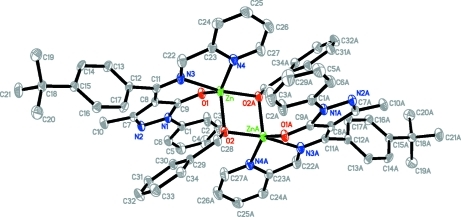
A view of the molecular structure of the title compound with displacement ellipsoids shown at the 20% probability level. Symmetry operator (A): 1-x,1-y,2-z.

## supplementary crystallographic information

### Comment

Polyesters such as poly(ε-caprolactone) (PCL) and poly(lactide) (PLA) and their copolymers have attracted intensive attention due to their potential applications in many fields (Gref *et al.*,1994; Jeong *et al.*, 1997). The major polymerization methods for these polymers are the ring-opening polymerization (ROP) of cyclic esters. Many zinc complexes with various ligands were effective initiator/catalyst for the ROP of cyclic esters (Chamberlain *et al.*, 2001; Williams *et al.*, 2003; Dechy-Cabaret *et al.* 2004, Chen, *et al.*, 2005; Wu, *et al.*, 2006; Hung *et al.*, 2008, 2009). Tripodal tridentate ligand supported zinc complexes have been synthesized and used for the polymerization of lactides and the polymerization is living with relatively low polydispersities (Chisholm *et al.*, 2000). Recently, a series of zinc alkoxides (Yu, *et al.*, 2002; Lee, *et al.*, 2007) coordinated with simple NNO-tridentate ketiminate ligands has been synthesized and these derivatives showed highly catalytic activity with regard to the ROP of lactides. However, these complexes had no (or little) stereoselectivities for the polymerization of *rac*-lactides. In order to obtain catalytic activity as well as enhance stereoselectivity of the corresponding metal alkoxides, we (Huang, *et al.*, 2009) have developed a unsymmetric NNO-ketiminate system derived from 4-benzoyl-3-methyl-1-phenyl-2-pyrazolin-5-one. In the presence of pyrazole fused a chelating arm, the metal derivatives can be stabilized by an extensive π-conjugate system. In this study, we reported the synthesis and crystal structure of [LZn(µ-OBn)]_2_, where *L* and benzyloxy are the NNO-tridentate ketiminate ligand and benzylalkoxy group, respectively.

This solid structure of [LZn(µ-OBn)]_2_ reveals a dimeric Zn^II^ complex in which the zinc center is coordinated to N,N,O atoms of the ketiminate ligand and two bridging oxygen atoms of the benzylalkoxy groups. The geometry around the zinc ion is a distorted trigonal bipyramid with τ = 0.51 and atoms O(1), O(2), and N(3) sitting on the equatorial positions. The bond distances between zinc and the coordinated atoms of Zn—O(2) 1.999 (2); Zn—O(1) 2.0137 (19); Zn—O(2 A) 2.0254 (17); Zn—N(3) 2.092 (2); and Zn—N(4) 2.115 (2) are compatible with the Zn—O and Zn—N distances found in other zinc ketiminate complexes (Huang *et al.*, 2009).

### Experimental

The ligand, (4*Z*)-4-{[(pyridin-2-yl)methylamino](4-*tert*-butylphenyl)methylene}-3-methyl-1-phenyl-1*H*-pyrazol-5(4*H*)-one (L—H) was prepared by the following procedures. 2-(Aminomethyl)pyridine (1.03 ml, 10.0 mmol) and 4-[(4-*tert*-butylphenyl)carbonyl]-3-methyl-1-phenyl-4,5-dihydro-1*H*-pyrazol-5-one (3.34 g, 10.0 mmol) were refluxed in ethanol (40 ml) for 24 h. The volatile materials were removed in under vacuum to produce yellow powder. The powder was then dissolved in hot ethanol (30 ml) and cooled to -18°C for 24 h giving yellow crystals.

The title complex was synthesized by the following procedures. Diethylzinc (2.2 ml, 1.0 *M* in hexane, 2.2 mmol) was slowly added to a suspension of L—H (0.85 g, 2.0 mmol) in toluene (40 ml). The mixture was stirred at 0°C for 3 h and the volatile materials were removed in under vacuum to yield white powder. The powder was dissolved in toluene (40 ml), and then benzyl alcohol (0.21 ml, 2.0 mmol) was added at 0°C. Continuously stirring at 0°C for another 3 h, the mixture initially turned colorless and then into white turbid. After filtration and washing with cooled toluene three times, a white powder was obtained. The resulting powder was recrystallized with a mixted dichloromathane and hexane solution to yield white crystals.

### Refinement

The methyl H atoms were  constrained to an ideal geometry with C—H distances of 0.96 Å and *U*_iso_(H) = 1.5*U*_eq_(C), but each group was allowed to rotate freely about its C—C bond. All other H atoms were placed in geometrically idealized positions and constrained to ride on their parent atoms with C—H distances in the range 0.95–1.00 Å and *U*_iso_(H) =1.2*U*_eq_(C).

### Crystal data

**Table:**

[Zn_2_(C_27_H_27_N_4_O)_2_(C_7_H_7_O)_2_]	*Z* = 1
*M**_r_* = 1192.04	*F*(000) = 624
Triclinic, *P*1	*D*_x_ = 1.332 Mg m^−^^3^
Hall symbol: -P 1	Mo *K*α radiation, λ = 0.71073 Å
*a* = 9.0873 (15) Å	Cell parameters from 4007 reflections
*b* = 13.363 (2) Å	θ = 2.3–26.0°
*c* = 13.397 (2) Å	µ = 0.86 mm^−^^1^
α = 72.206 (3)°	*T* = 293 K
β = 74.018 (3)°	Parallelepiped, white
γ = 88.664 (3)°	0.41 × 0.32 × 0.25 mm
*V* = 1485.9 (4) Å^3^	

### Data collection

**Table:**

Bruker SMART 1000 CCD diffractometer	5792 independent reflections
Radiation source: fine-focus sealed tube	4450 reflections with *I* > 2σ(*I*)
graphite	*R*_int_ = 0.031
φ and ω scans	θ_max_ = 26.1°, θ_min_ = 1.7°
Absorption correction: multi-scan (*SADABS*; Sheldrick, 1996)	*h* = −11→11
*T*_min_ = 0.719, *T*_max_ = 0.813	*k* = −16→14
8258 measured reflections	*l* = −16→16

### Refinement

**Table:**

Refinement on *F*^2^	Primary atom site location: structure-invariant direct methods
Least-squares matrix: full	Secondary atom site location: difference Fourier map
*R*[*F*^2^ > 2σ(*F*^2^)] = 0.048	Hydrogen site location: inferred from neighbouring sites
*wR*(*F*^2^) = 0.137	H-atom parameters constrained
*S* = 1.01	*w* = 1/[σ^2^(*F*_o_^2^) + (0.0902*P*)^2^] where *P* = (*F*_o_^2^ + 2*F*_c_^2^)/3
5792 reflections	(Δ/σ)_max_ = 0.001
370 parameters	Δρ_max_ = 0.58 e Å^−^^3^
0 restraints	Δρ_min_ = −0.44 e Å^−^^3^

### Special details

**Table:**

Geometry. All e.s.d.'s (except the e.s.d. in the dihedral angle between two l.s. planes) are estimated using the full covariance matrix. The cell e.s.d.'s are taken into account individually in the estimation of e.s.d.'s in distances, angles and torsion angles; correlations between e.s.d.'s in cell parameters are only used when they are defined by crystal symmetry. An approximate (isotropic) treatment of cell e.s.d.'s is used for estimating e.s.d.'s involving l.s. planes.
Refinement. Refinement of *F*^2^ against ALL reflections. The weighted *R*-factor *wR* and goodness of fit *S* are based on *F*^2^, conventional *R*-factors *R* are based on *F*, with *F* set to zero for negative *F*^2^. The threshold expression of *F*^2^ > σ(*F*^2^) is used only for calculating *R*-factors(gt) *etc*. and is not relevant to the choice of reflections for refinement. *R*-factors based on *F*^2^ are statistically about twice as large as those based on *F*, and *R*- factors based on ALL data will be even larger.

### Fractional atomic coordinates and isotropic or equivalent isotropic displacement parameters (Å^2^)

**Table:**

	*x*	*y*	*z*	*U*_iso_*/*U*_eq_	
Zn	0.65973 (4)	0.51056 (2)	0.92949 (2)	0.04217 (14)	
O1	0.6604 (2)	0.39430 (15)	0.86285 (14)	0.0471 (5)	
O2	0.4651 (2)	0.58125 (14)	0.92139 (14)	0.0427 (5)	
N1	0.5943 (3)	0.36547 (18)	0.71795 (17)	0.0414 (5)	
N2	0.6169 (3)	0.41391 (19)	0.60618 (18)	0.0479 (6)	
N3	0.8186 (3)	0.5971 (2)	0.78379 (19)	0.0502 (6)	
N4	0.8045 (3)	0.5874 (2)	0.98662 (19)	0.0510 (6)	
C1	0.4996 (3)	0.2706 (2)	0.7697 (2)	0.0445 (7)	
C2	0.4698 (5)	0.2198 (3)	0.8796 (3)	0.0766 (12)	
H2B	0.5121	0.2468	0.9226	0.092*	
C3	0.3759 (6)	0.1277 (4)	0.9260 (3)	0.0979 (17)	
H3A	0.3572	0.0926	1.0003	0.118*	
C4	0.3106 (5)	0.0875 (3)	0.8657 (3)	0.0866 (13)	
H4A	0.2459	0.0266	0.8984	0.104*	
C5	0.3417 (5)	0.1382 (3)	0.7558 (3)	0.0741 (11)	
H5A	0.2988	0.1109	0.7133	0.089*	
C6	0.4359 (4)	0.2295 (3)	0.7076 (3)	0.0557 (8)	
H6A	0.4563	0.2633	0.6329	0.067*	
C7	0.7005 (3)	0.5016 (2)	0.5823 (2)	0.0420 (6)	
C8	0.7365 (3)	0.5136 (2)	0.6750 (2)	0.0397 (6)	
C9	0.6646 (3)	0.4231 (2)	0.7623 (2)	0.0388 (6)	
C10	0.7436 (4)	0.5707 (3)	0.4660 (2)	0.0585 (8)	
H10A	0.7002	0.5397	0.4238	0.088*	
H10B	0.8533	0.5779	0.4373	0.088*	
H10C	0.7050	0.6388	0.4624	0.088*	
C11	0.8166 (3)	0.5975 (2)	0.6870 (2)	0.0401 (6)	
C12	0.8939 (3)	0.6863 (2)	0.5875 (2)	0.0415 (6)	
C13	1.0346 (4)	0.6780 (2)	0.5185 (2)	0.0527 (8)	
H13A	1.0860	0.6169	0.5355	0.063*	
C14	1.0994 (4)	0.7592 (3)	0.4250 (2)	0.0543 (8)	
H14A	1.1948	0.7519	0.3803	0.065*	
C15	1.0275 (3)	0.8518 (2)	0.3950 (2)	0.0449 (7)	
C16	0.8896 (4)	0.8605 (2)	0.4664 (2)	0.0507 (7)	
H16A	0.8394	0.9224	0.4506	0.061*	
C17	0.8241 (3)	0.7793 (2)	0.5609 (2)	0.0488 (7)	
H17A	0.7310	0.7878	0.6074	0.059*	
C18	1.1010 (4)	0.9370 (3)	0.2872 (2)	0.0528 (8)	
C19	1.2569 (4)	0.9755 (3)	0.2883 (3)	0.0788 (12)	
H19A	1.2437	1.0052	0.3468	0.118*	
H19B	1.3040	1.0282	0.2200	0.118*	
H19C	1.3212	0.9174	0.2989	0.118*	
C20	1.0036 (5)	1.0308 (3)	0.2664 (3)	0.0740 (11)	
H20A	0.9903	1.0621	0.3237	0.111*	
H20B	0.9052	1.0079	0.2648	0.111*	
H20C	1.0537	1.0818	0.1975	0.111*	
C21	1.1214 (5)	0.8895 (3)	0.1935 (3)	0.0781 (12)	
H21A	1.0231	0.8645	0.1937	0.117*	
H21B	1.1866	0.8319	0.2033	0.117*	
H21C	1.1670	0.9424	0.1250	0.117*	
C22	0.9139 (4)	0.6774 (3)	0.7947 (3)	0.0656 (10)	
H22A	0.8742	0.7459	0.7704	0.079*	
H22B	1.0177	0.6796	0.7487	0.079*	
C23	0.9158 (3)	0.6541 (2)	0.9110 (2)	0.0471 (7)	
C24	1.0260 (4)	0.7008 (3)	0.9383 (3)	0.0596 (8)	
H24A	1.1038	0.7462	0.8838	0.072*	
C25	1.0206 (4)	0.6803 (3)	1.0458 (3)	0.0723 (11)	
H25A	1.0943	0.7111	1.0655	0.087*	
C26	0.9044 (5)	0.6135 (4)	1.1235 (3)	0.0837 (13)	
H26A	0.8966	0.5987	1.1973	0.100*	
C27	0.8007 (5)	0.5690 (3)	1.0911 (3)	0.0770 (12)	
H27A	0.7226	0.5232	1.1446	0.092*	
C28	0.4663 (4)	0.6915 (2)	0.8848 (2)	0.0480 (7)	
H28A	0.5652	0.7207	0.8809	0.058*	
H28B	0.3889	0.7142	0.9380	0.058*	
C29	0.4367 (3)	0.7360 (2)	0.7741 (2)	0.0403 (6)	
C30	0.4252 (3)	0.6741 (2)	0.7107 (2)	0.0487 (7)	
H30A	0.4371	0.6022	0.7354	0.058*	
C31	0.3960 (4)	0.7178 (3)	0.6106 (3)	0.0654 (10)	
H31A	0.3850	0.6749	0.5697	0.078*	
C32	0.3834 (5)	0.8241 (4)	0.5718 (3)	0.0781 (12)	
H32A	0.3656	0.8536	0.5041	0.094*	
C33	0.3972 (5)	0.8868 (3)	0.6333 (3)	0.0729 (11)	
H33A	0.3891	0.9590	0.6069	0.087*	
C34	0.4229 (4)	0.8433 (3)	0.7342 (2)	0.0534 (8)	
H34A	0.4310	0.8864	0.7756	0.064*	

### Atomic displacement parameters (Å^2^)

**Table:**

	*U*^11^	*U*^22^	*U*^33^	*U*^12^	*U*^13^	*U*^23^
Zn	0.0478 (2)	0.0436 (2)	0.02763 (18)	−0.01468 (14)	−0.00437 (13)	−0.00471 (13)
O1	0.0647 (13)	0.0423 (11)	0.0272 (9)	−0.0156 (9)	−0.0091 (9)	−0.0030 (8)
O2	0.0523 (12)	0.0410 (11)	0.0266 (9)	−0.0093 (9)	−0.0090 (8)	0.0000 (8)
N1	0.0531 (14)	0.0391 (12)	0.0267 (11)	−0.0090 (10)	−0.0062 (10)	−0.0066 (9)
N2	0.0598 (16)	0.0497 (14)	0.0302 (12)	−0.0059 (12)	−0.0118 (11)	−0.0069 (10)
N3	0.0580 (15)	0.0508 (14)	0.0328 (12)	−0.0232 (12)	−0.0025 (11)	−0.0077 (11)
N4	0.0551 (15)	0.0541 (15)	0.0392 (13)	−0.0164 (12)	−0.0099 (11)	−0.0095 (11)
C1	0.0483 (16)	0.0417 (15)	0.0385 (15)	−0.0071 (12)	−0.0060 (12)	−0.0101 (12)
C2	0.108 (3)	0.074 (2)	0.0359 (17)	−0.049 (2)	−0.0090 (18)	−0.0065 (16)
C3	0.137 (4)	0.088 (3)	0.046 (2)	−0.065 (3)	−0.010 (2)	0.001 (2)
C4	0.095 (3)	0.078 (3)	0.072 (3)	−0.050 (2)	−0.009 (2)	−0.013 (2)
C5	0.082 (3)	0.073 (2)	0.069 (2)	−0.027 (2)	−0.025 (2)	−0.019 (2)
C6	0.066 (2)	0.0536 (18)	0.0472 (17)	−0.0116 (15)	−0.0185 (15)	−0.0125 (15)
C7	0.0477 (16)	0.0404 (15)	0.0321 (13)	−0.0004 (12)	−0.0072 (12)	−0.0063 (12)
C8	0.0460 (15)	0.0356 (14)	0.0291 (13)	−0.0032 (11)	−0.0034 (11)	−0.0042 (11)
C9	0.0429 (15)	0.0360 (14)	0.0320 (13)	−0.0025 (11)	−0.0036 (11)	−0.0084 (11)
C10	0.081 (2)	0.0513 (18)	0.0347 (15)	−0.0091 (16)	−0.0180 (15)	0.0010 (14)
C11	0.0387 (14)	0.0379 (14)	0.0339 (14)	−0.0030 (11)	−0.0021 (11)	−0.0042 (11)
C12	0.0433 (15)	0.0399 (15)	0.0323 (13)	−0.0091 (12)	−0.0024 (11)	−0.0050 (11)
C13	0.0492 (17)	0.0459 (17)	0.0440 (16)	0.0024 (13)	0.0039 (13)	−0.0022 (14)
C14	0.0409 (16)	0.0532 (18)	0.0461 (17)	0.0005 (13)	0.0096 (13)	−0.0025 (14)
C15	0.0422 (15)	0.0438 (16)	0.0377 (15)	−0.0102 (12)	−0.0051 (12)	−0.0016 (12)
C16	0.0544 (18)	0.0388 (16)	0.0455 (17)	−0.0009 (13)	−0.0050 (14)	−0.0018 (13)
C17	0.0427 (16)	0.0471 (17)	0.0430 (16)	−0.0017 (13)	0.0042 (13)	−0.0088 (13)
C18	0.0507 (17)	0.0523 (18)	0.0417 (16)	−0.0163 (14)	−0.0078 (13)	0.0017 (14)
C19	0.060 (2)	0.083 (3)	0.066 (2)	−0.034 (2)	−0.0106 (18)	0.013 (2)
C20	0.077 (3)	0.053 (2)	0.067 (2)	−0.0181 (18)	−0.015 (2)	0.0134 (18)
C21	0.094 (3)	0.081 (3)	0.0379 (18)	−0.019 (2)	−0.0038 (18)	0.0005 (18)
C22	0.077 (2)	0.063 (2)	0.0444 (17)	−0.0382 (18)	−0.0040 (16)	−0.0076 (16)
C23	0.0465 (16)	0.0455 (16)	0.0438 (16)	−0.0097 (13)	−0.0053 (13)	−0.0118 (13)
C24	0.0512 (19)	0.061 (2)	0.063 (2)	−0.0147 (15)	−0.0099 (16)	−0.0178 (17)
C25	0.068 (2)	0.086 (3)	0.073 (2)	−0.013 (2)	−0.034 (2)	−0.026 (2)
C26	0.100 (3)	0.102 (3)	0.053 (2)	−0.030 (3)	−0.032 (2)	−0.018 (2)
C27	0.091 (3)	0.091 (3)	0.0382 (18)	−0.039 (2)	−0.0133 (18)	−0.0074 (18)
C28	0.0621 (19)	0.0452 (16)	0.0309 (14)	−0.0088 (14)	−0.0118 (13)	−0.0040 (12)
C29	0.0336 (14)	0.0458 (15)	0.0309 (13)	−0.0058 (11)	−0.0021 (11)	−0.0025 (11)
C30	0.0511 (17)	0.0510 (17)	0.0347 (14)	−0.0074 (13)	−0.0073 (13)	−0.0037 (13)
C31	0.073 (2)	0.082 (3)	0.0418 (18)	−0.0075 (19)	−0.0205 (17)	−0.0154 (18)
C32	0.088 (3)	0.095 (3)	0.045 (2)	0.007 (2)	−0.032 (2)	0.000 (2)
C33	0.088 (3)	0.057 (2)	0.055 (2)	0.0158 (19)	−0.0182 (19)	0.0073 (17)
C34	0.0531 (18)	0.0544 (19)	0.0442 (17)	0.0009 (14)	−0.0103 (14)	−0.0062 (14)

### Geometric parameters (Å, °)

**Table:**

Zn—O2	1.999 (2)	C15—C16	1.379 (4)
Zn—O1	2.0137 (19)	C15—C18	1.526 (4)
Zn—O2^i^	2.0253 (17)	C16—C17	1.382 (4)
Zn—N3	2.092 (2)	C16—H16A	0.9300
Zn—N4	2.114 (2)	C17—H17A	0.9300
Zn—Zn^i^	2.9612 (8)	C18—C20	1.518 (5)
O1—C9	1.272 (3)	C18—C19	1.523 (5)
O2—C28	1.403 (3)	C18—C21	1.539 (5)
O2—Zn^i^	2.0253 (17)	C19—H19A	0.9600
N1—C9	1.367 (3)	C19—H19B	0.9600
N1—N2	1.396 (3)	C19—H19C	0.9600
N1—C1	1.419 (3)	C20—H20A	0.9600
N2—C7	1.315 (4)	C20—H20B	0.9600
N3—C11	1.300 (4)	C20—H20C	0.9600
N3—C22	1.460 (4)	C21—H21A	0.9600
N4—C23	1.329 (4)	C21—H21B	0.9600
N4—C27	1.335 (4)	C21—H21C	0.9600
C1—C2	1.372 (4)	C22—C23	1.498 (4)
C1—C6	1.374 (4)	C22—H22A	0.9700
C2—C3	1.384 (5)	C22—H22B	0.9700
C2—H2B	0.9300	C23—C24	1.377 (4)
C3—C4	1.355 (5)	C24—C25	1.368 (5)
C3—H3A	0.9300	C24—H24A	0.9300
C4—C5	1.370 (5)	C25—C26	1.366 (5)
C4—H4A	0.9300	C25—H25A	0.9300
C5—C6	1.378 (5)	C26—C27	1.356 (5)
C5—H5A	0.9300	C26—H26A	0.9300
C6—H6A	0.9300	C27—H27A	0.9300
C7—C8	1.423 (4)	C28—C29	1.516 (4)
C7—C10	1.495 (4)	C28—H28A	0.9700
C8—C9	1.418 (4)	C28—H28B	0.9700
C8—C11	1.422 (4)	C29—C30	1.378 (4)
C10—H10A	0.9600	C29—C34	1.385 (4)
C10—H10B	0.9600	C30—C31	1.386 (4)
C10—H10C	0.9600	C30—H30A	0.9300
C11—C12	1.495 (3)	C31—C32	1.369 (6)
C12—C17	1.375 (4)	C31—H31A	0.9300
C12—C13	1.381 (4)	C32—C33	1.370 (6)
C13—C14	1.374 (4)	C32—H32A	0.9300
C13—H13A	0.9300	C33—C34	1.380 (5)
C14—C15	1.386 (4)	C33—H33A	0.9300
C14—H14A	0.9300	C34—H34A	0.9300
			
O2—Zn—O1	105.69 (8)	C14—C15—C18	119.9 (3)
O2—Zn—O2^i^	85.26 (8)	C15—C16—C17	121.4 (3)
O1—Zn—O2^i^	92.46 (7)	C15—C16—H16A	119.3
O2—Zn—N3	103.26 (9)	C17—C16—H16A	119.3
O1—Zn—N3	87.87 (9)	C12—C17—C16	121.3 (3)
O2^i^—Zn—N3	171.07 (9)	C12—C17—H17A	119.3
O2—Zn—N4	113.40 (10)	C16—C17—H17A	119.3
O1—Zn—N4	140.49 (10)	C15—C18—C20	112.5 (3)
O2^i^—Zn—N4	96.22 (8)	C15—C18—C19	109.5 (3)
N3—Zn—N4	78.03 (9)	C20—C18—C19	108.5 (3)
O2—Zn—Zn^i^	42.97 (5)	C15—C18—C21	108.6 (3)
O1—Zn—Zn^i^	102.23 (6)	C20—C18—C21	108.1 (3)
O2^i^—Zn—Zn^i^	42.29 (6)	C19—C18—C21	109.7 (3)
N3—Zn—Zn^i^	146.15 (8)	C18—C19—H19A	109.5
N4—Zn—Zn^i^	110.01 (7)	C18—C19—H19B	109.5
C9—O1—Zn	116.06 (17)	H19A—C19—H19B	109.5
C28—O2—Zn	120.26 (18)	C18—C19—H19C	109.5
C28—O2—Zn^i^	124.18 (17)	H19A—C19—H19C	109.5
Zn—O2—Zn^i^	94.74 (8)	H19B—C19—H19C	109.5
C9—N1—N2	111.9 (2)	C18—C20—H20A	109.5
C9—N1—C1	129.6 (2)	C18—C20—H20B	109.5
N2—N1—C1	118.4 (2)	H20A—C20—H20B	109.5
C7—N2—N1	105.2 (2)	C18—C20—H20C	109.5
C11—N3—C22	119.5 (2)	H20A—C20—H20C	109.5
C11—N3—Zn	124.61 (19)	H20B—C20—H20C	109.5
C22—N3—Zn	114.56 (19)	C18—C21—H21A	109.5
C23—N4—C27	117.6 (3)	C18—C21—H21B	109.5
C23—N4—Zn	116.6 (2)	H21A—C21—H21B	109.5
C27—N4—Zn	125.7 (2)	C18—C21—H21C	109.5
C2—C1—C6	119.6 (3)	H21A—C21—H21C	109.5
C2—C1—N1	121.6 (3)	H21B—C21—H21C	109.5
C6—C1—N1	118.8 (3)	N3—C22—C23	110.6 (2)
C1—C2—C3	119.2 (3)	N3—C22—H22A	109.5
C1—C2—H2B	120.4	C23—C22—H22A	109.5
C3—C2—H2B	120.4	N3—C22—H22B	109.5
C4—C3—C2	121.6 (4)	C23—C22—H22B	109.5
C4—C3—H3A	119.2	H22A—C22—H22B	108.1
C2—C3—H3A	119.2	N4—C23—C24	121.7 (3)
C3—C4—C5	118.9 (3)	N4—C23—C22	116.7 (3)
C3—C4—H4A	120.5	C24—C23—C22	121.5 (3)
C5—C4—H4A	120.5	C23—C24—C25	119.7 (3)
C4—C5—C6	120.5 (3)	C23—C24—H24A	120.1
C4—C5—H5A	119.7	C25—C24—H24A	120.1
C6—C5—H5A	119.7	C26—C25—C24	118.5 (3)
C1—C6—C5	120.2 (3)	C26—C25—H25A	120.8
C1—C6—H6A	119.9	C24—C25—H25A	120.8
C5—C6—H6A	119.9	C27—C26—C25	118.9 (3)
N2—C7—C8	112.4 (2)	C27—C26—H26A	120.6
N2—C7—C10	117.1 (3)	C25—C26—H26A	120.6
C8—C7—C10	130.5 (3)	N4—C27—C26	123.6 (3)
C7—C8—C9	104.7 (2)	N4—C27—H27A	118.2
C7—C8—C11	131.0 (2)	C26—C27—H27A	118.2
C9—C8—C11	124.2 (2)	O2—C28—C29	114.0 (2)
O1—C9—N1	123.1 (2)	O2—C28—H28A	108.7
O1—C9—C8	131.0 (3)	C29—C28—H28A	108.7
N1—C9—C8	105.8 (2)	O2—C28—H28B	108.7
C7—C10—H10A	109.5	C29—C28—H28B	108.7
C7—C10—H10B	109.5	H28A—C28—H28B	107.6
H10A—C10—H10B	109.5	C30—C29—C34	118.5 (3)
C7—C10—H10C	109.5	C30—C29—C28	122.6 (3)
H10A—C10—H10C	109.5	C34—C29—C28	118.8 (3)
H10B—C10—H10C	109.5	C29—C30—C31	120.7 (3)
N3—C11—C8	119.8 (2)	C29—C30—H30A	119.7
N3—C11—C12	121.1 (3)	C31—C30—H30A	119.7
C8—C11—C12	119.0 (2)	C32—C31—C30	120.2 (4)
C17—C12—C13	117.7 (3)	C32—C31—H31A	119.9
C17—C12—C11	120.3 (3)	C30—C31—H31A	119.9
C13—C12—C11	122.0 (3)	C31—C32—C33	119.7 (3)
C14—C13—C12	120.7 (3)	C31—C32—H32A	120.2
C14—C13—H13A	119.7	C33—C32—H32A	120.2
C12—C13—H13A	119.7	C32—C33—C34	120.4 (3)
C13—C14—C15	122.2 (3)	C32—C33—H33A	119.8
C13—C14—H14A	118.9	C34—C33—H33A	119.8
C15—C14—H14A	118.9	C33—C34—C29	120.5 (3)
C16—C15—C14	116.6 (3)	C33—C34—H34A	119.8
C16—C15—C18	123.5 (3)	C29—C34—H34A	119.8

Symmetry codes: (i) −*x*+1, −*y*+1, −*z*+2.
